# The Clinical Understaging of Recurrent Glottic Carcinoma after Radiation Failure

**DOI:** 10.1155/2016/2706463

**Published:** 2016-02-18

**Authors:** Moustafa Mourad, Sami P. Moubayed, Ilya Likhterov, Mark Urken

**Affiliations:** Department of Otolaryngology-Head and Neck Surgery, Mount Sinai Beth Israel, New York, NY 10003, USA

## Abstract

*Background.* Recurrent glottic squamous cell carcinomas following radiation therapy for early staged tumors are oftentime early staged tumors. Management of these early stage recurrences presents a dilemma for the head and neck surgeon. Difficulties in appropriate tumor mapping, preoperative analysis, and poor understanding of the virulent pathologic nature of the recurrence may impede surgical decision-making.* Methods.* This is a single surgeon case report, presenting a patient with rapid recurrence following salvage transoral resection for an early stage recurrence, necessitating a total laryngectomy.* Results.* A review of the literature was performed, identifying studies that expound on the pathologic behavior of radiation recurrent disease.* Conclusions.* Radiation recurrent glottic squamous cell carcinoma has a distinct pathologic behavior and aggressive nature. Disease virulence, coupled with difficulty in appropriate staging and preoperative tumor mapping, should guide the surgeon when deciding the surgical management in the salvage setting.

## 1. Introduction

The failure rates of early stage glottic squamous cell carcinoma (SCC) treated with radiation therapy as a single modality can reach 5–13% for T1 tumors and 25–30% for T2 tumors [1]. The traditional standard of care for recurrent or persistent glottic SCC is a total laryngectomy, although more and more authors favor partial laryngectomy (endoscopic or open) in a carefully selected subset of early stage recurrences [2]. Unfortunately, the clinical staging of these cancers is challenging due to postradiation fibrosis and edema and a different, multicentric, tumor growth pattern [3]. We present a case of recurrent glottic SCC that was clinically and radiographically understaged and a discussion with potential solutions to avoid this problem. This report was approved by the institutional review board (Program for the Protection of Human Subjects of Icahn School of Medicine at Mount Sinai).

## 2. Case Report

**Figure 1:**
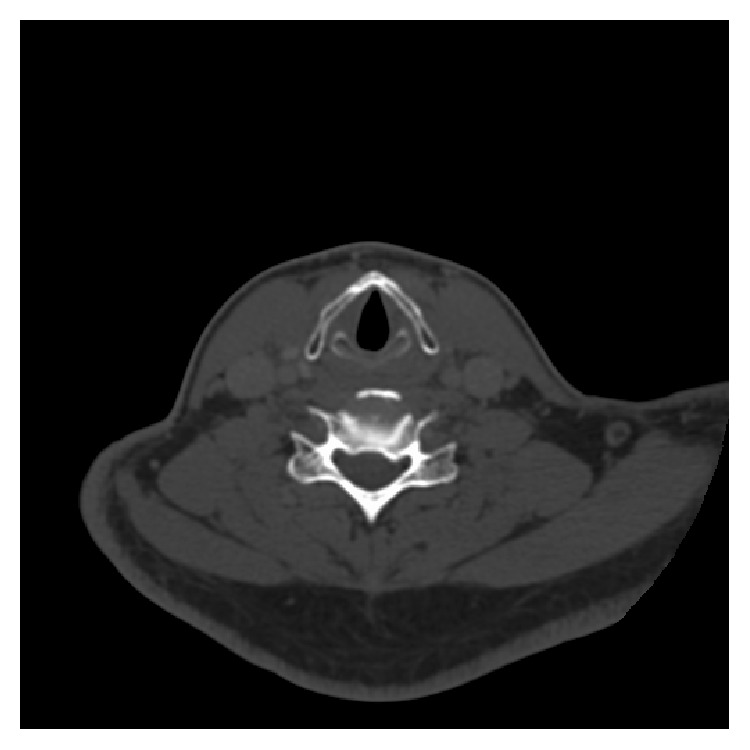
Axial computed tomography image of radiation recurrent glottic cancer shows no evidence of invasive disease into the thyroid cartilage prior to initial salvage surgery.

**Figure 2:**
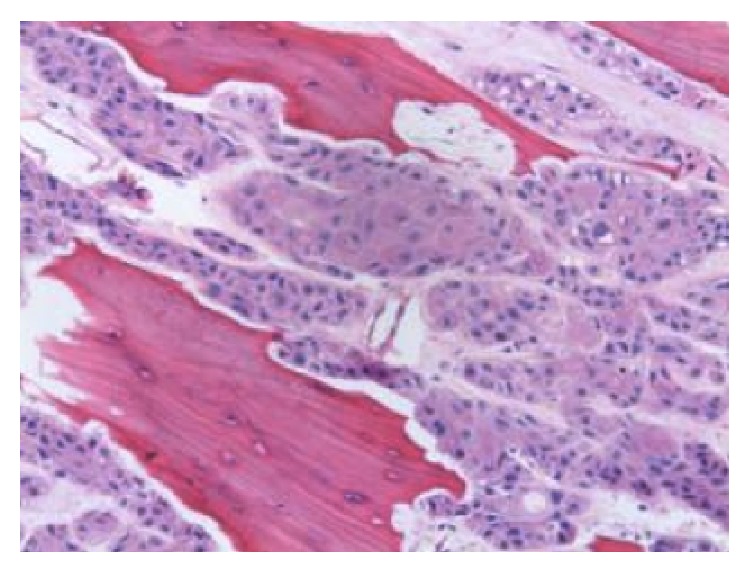
Low magnification (20x) hematoxylin and eosin pathological photomicrograph showing squamous cell carcinoma invading into ossified cartilage.

A 47-year-old male was treated with a salvage vertical partial hemilaryngectomy after failed radiation for an early stage (cT1a) squamous cell carcinoma of the glottis. The recurrent tumor was initially staged as rT1a with normal vocal cord mobility (Figure 1). Although surgical margins were free of disease, the lesion was found to invade the thyroid cartilage on final pathology, prompting it to be restaged as rT4a (Figure 2). The patient subsequently rapidly developed extensive disease involving the subglottis with extension into the overlying strap muscles and underwent a salvage total laryngectomy. The patient was free from disease 24 months after laryngectomy.

## 3. Discussion

The important clinicopathologic features of the presented patient highlight the fact that a recurrent laryngeal SCC may be clinically understaged. The reasons for this have been outlined in pathological studies [3], which have shown that, compared with primary disease of the same stage, recurrent glottic SCC demonstrates (1) multifocal growth, including extralaryngeal sites, (2) a higher percentage of undifferentiated tumor cells, (3) increased perineural invasion, (4) increased cartilage invasion, (5) increased contralateral tumor involvement, (6) increased spread along mucous glands and blood vessels, and (7) increased propensity for subglottic spread [3]. When taken in aggregate, these factors make tumor mapping and staging increasingly difficult. As a result of the highlighted differences in tumor biology, although a third of glottic SCC recurrences are early stage (T1 or T2) recurrences, up to 52% of these are understaged clinically based on imaging and endoscopy [3]. This study however did not distinguish between CT and MRI as to the preferred imaging modalities. Disease extent may be difficult to distinguish from posttreatment radiographic changes. In addition, vocal cord mobility may be hindered by radiation effects or through paraglottic space involvement by the tumor.

Accurate assessment of the paraglottic space is critical when considering surgical organ preservation strategies. The traditional contraindications for partial laryngeal surgery are contralateral vocal cord involvement, arytenoid involvement (except for vocal process), subglottic extension greater than 5 mm, cartilage involvement, and vocal cord fixation [4].

Appropriate preoperative staging is paramount. Cartilage biopsies carry a high risk of chondronecrosis and are inadvisable [5]. There is limited literature on the ideal imaging test to assess recurrent or persistent laryngeal SCC. Current data, which includes some systematic reviews [6], shows that neither CT (sensitivity of 57% and specificity of 94%) nor MRI (sensitivity of 72% and specificity of 57%) can effectively and consistently distinguish postradiation changes from cartilage invasion. PET-CT has improved sensitivity of 89% and specificity of 74%, while diffusion-weighted MRI has a reported sensitivity of 94% and specificity of 100%. Still these modalities are not as widely utilized and the data is based on limited clinical experience [6]. Finally, it is also possible for a tumor to progress between the time of radiological evaluation and surgery, as one month had elapsed in our case between the CT scan and the partial laryngectomy.

We have described a two-staged laryngeal reconstruction technique after vertical partial laryngectomy, which creates a temporary laryngostome with turn-in flaps following resection, the creation of a titanium-mesh composite prefabricated flap during the second stage, and closure during the third stage [7]. This allows definitive reconstruction to be deferred until verification on permanent section has proven negative margins and evaluated the growth pattern of the recurrent disease [7]. Laryngectomy is held in reserve for positive margins.

Recurrent or persistent glottic SCC must be carefully evaluated with endoscopy, PET-CT, and diffusion-weighted MRI, if possible [6].
